# Cerebrovascular Accident Risk and Guidewire Choice in Transcatheter Aortic Valve Replacement Procedure

**DOI:** 10.7759/cureus.105140

**Published:** 2026-03-12

**Authors:** Muhammad Khan, Yashaswi Joshi, Zachary Spires, Kirsten Ferraro, Stefan Weston, Parth Patel, Aladen Amro, Robert E Heidel, Raj Baljepally

**Affiliations:** 1 Cardiovascular Medicine, University of Tennessee Medical Center, Knoxville, USA; 2 Internal Medicine, University of Tennessee Medical Center, Knoxville, USA; 3 Biostatistics, University of Tennessee Health Science Center College of Medicine, Knoxville, Knoxville, USA

**Keywords:** cerebrovascular accidents (cva), embolic protection device, permanent pacemaker implantation (ppm), post tavr copmlications, severe aortic stenosis

## Abstract

Introduction: Transcatheter aortic valve replacement (TAVR) has become an established minimally invasive treatment for patients with severe symptomatic aortic stenosis (AS), particularly those at high surgical risk. One procedural step involves advancing a guidewire across the aortic valve (AV) into the left ventricle (LV). Prior studies have shown that manipulation of calcified structures, vascular plaque embolization, and prolonged procedural time may increase the risk of cerebrovascular accidents (CVA) associated with TAVR. This study aimed to compare the incidence of CVA and other periprocedural outcomes based on the type of guidewire used to cross the AV during TAVR.

Methods: We conducted a retrospective analysis of 537 patients who had undergone TAVR for symptomatic severe AS between July 2018 and July 2023 at our healthcare facility. Patients were divided into two groups: those receiving a 0.035” hydrophilic guidewire (n = 253) versus a 0.038” fiber-core straight TEF guidewire (n = 284) for AV crossing. Baseline demographics, procedural characteristics, and outcomes were compared between groups. The primary outcomes were CVA within 24 hours and within one month. Secondary outcomes included permanent pacemaker (PPM) implantation, myocardial infarction (MI), and all-cause mortality at one month.

Results: There were no statistically significant differences in the incidence of CVA between the hydrophilic and fiber-core wire groups at 24 hours (p = 0.71) or one month (p = 0.43). Similarly, there were no significant differences in PPM implantation at 24-48 hours (p = 0.23) or at one month (p = 0.42), in one-month all-cause mortality (p = 0.29), or in MI within one month (p = 1.00).

Conclusion: The type of guidewire used to cross the AV during TAVR, whether 0.035” hydrophilic or 0.038” fiber-core straight TEF, was not associated with significant differences in the incidence of CVA or other major adverse clinical outcomes. These findings suggest that guidewire selection for AV crossing may be based on operator preference without compromising cerebrovascular safety.

## Introduction

Pathophysiology of aortic stenosis (AS) includes initial endothelial injury and dysfunction, followed by calcification. This stiffens the aortic valve (AV) cusps, and a reduction in valve opening ensues. Transcatheter AV replacement (TAVR) is a minimally invasive technique for treating severe AS. It involves inserting a bioprosthetic valve via a catheter and implanting it within the diseased native AV. It is superior to standard medical therapy only in inoperable patients with a life expectancy of more than one year. It received a Class 1 recommendation from the American College of Cardiology/American Heart Association (ACC/AHA) for patients over 80 years of age or for younger patients with a life expectancy of less than 10 years and no anatomic contraindication to TAVR [1]. Cerebrovascular accident (CVA) is a well-recognized complication that can occur during or after TAVR [2]. Although the risk of CVA was relatively high in the early TAVR experience, recent advances such as the development of less traumatic delivery catheters, improved procedural techniques, and better patient selection have contributed to a decline in stroke incidence [3]. Nevertheless, current TAVR series still reports a 1% to 4% risk of clinically apparent stroke, which remains associated with significant morbidity and mortality [3].

Multiple studies have identified procedural risk factors that contribute to CVA, including prolonged catheter manipulation, extensive AV and left ventricular outflow tract (LVOT) calcification, and prosthetic valve deployment [4]. One of the key mechanisms of stroke during TAVR is cerebral embolization of calcified material, vascular plaque, and tissue debris originating from the AV, aortic arch, or left ventricle [5,6].

In this study, we aimed to determine whether the type of guidewire used to cross the AV affects the incidence of CVA. Specifically, we compared a 0.035” hydrophilic guidewire with a 0.038” fiber-core straight TEF guidewire (non-hydrophilic) during left ventricular cavity access in the TAVR procedure. We evaluated the incidence of CVA at 24 hours and at one-month follow-up. Additionally, we assessed other common TAVR-related complications, including PPM (permanent pacemaker) implantation and all-cause mortality.

## Materials and methods

In this single-center study, we conducted a retrospective analysis of 537 patients who had undergone TAVR for symptomatic severe AS between July 2018 and July 2023 at our healthcare facility. Based on the operator's preference for guidewire use, patients were divided into two groups: those receiving a 0.035” hydrophilic guidewire (n = 253) versus a 0.038” fiber-core straight TEF guidewire (n = 284) for AV crossing. Baseline demographics, procedural characteristics, and outcomes were compared between groups. The primary outcomes included CVA within 24 hours and within one month of the procedure. Secondary outcomes included PPM implantation, myocardial infarction (MI), and all-cause mortality at one month.

Statistical methods

Frequency and descriptive statistics were performed to describe the demographic and clinical characteristics of the sample groups (hydrophilic 0.035 (Hydro) vs. FC straight TEF 0.038 (FC)). The statistical assumptions of normality (Shapiro-Wilk test, histograms, QQ plot) and homogeneity of variance (Levene’s Test of Equality of Variances) were tested before performing parametric analyses. Age was compared between the two guidewire groups using an unpaired t-test, and the average age for the two groups was reported along with their respective standard deviations. All other parameters in the study were categorical, and the guidewire groups were compared using either chi-square analysis (gender, dyslipidemia, hypertension, diabetes, chronic kidney disease, smoking, obstructive sleep apnea, left ventricular ejection fraction, history of thrombophilia disorder, history of coronary artery bypass graft, history of native coronary artery disease, PPM need in one month), Fisher’s exact test (CVA during or within 24 hours, CVA within one month of procedure, PPM need within 24-48 hours, one-month all-cause mortality, MI within one month of TAVR), or likelihood ratios (ethnicity and BMI). Frequency and percentage statistics were reported and interpreted for the categorical analyses. No formal adjustment for multiple comparisons was performed, as the analyses were employed to test for exploratory, unadjusted associations across a wide range of pertinent demographic and clinical variables versus attempting to proffer causal inferences. Furthermore, adjustments to alpha values could have increased the risk of Type II errors given the low event rates and correlated clinical variables. Statistical significance was assumed at a two-sided alpha value of 0.05, and all analyses were conducted using IBM SPSS Statistics for Windows, Version 29 (Released 2023; IBM Corp., Armonk, New York).

## Results

The frequency and descriptive statistics for the guidewire group comparisons are presented in Table 1. The Hydrophilic group had a significantly higher age, t (535) = 2.94, p = 0.003. No differences were detected between the groups in any other demographic or baseline clinical parameters (p > 0.05). In terms of primary outcomes, there were no statistically significant differences in the incidence of CVA at 24 hours (Hydro (n = 4, 1.6%) vs. FC (n = 3, 1.1%), X² (1, n = 537) = 0.29, p = 0.71) or one month (Hydro (n = 4, 1.6%) vs. FC (n = 2, 0.7%), X² (1, n = 535) = 0.93, p = 0.43). No effect was detected for PPM implantation at 24-48 hours (Hydro (n = 6, 2.4%) vs. FC (n = 3, 1.1%), X² (1, n = 531) = 1.44, p = 0.32) or one month (Hydro (n = 7, 2.8%) vs. FC (n = 5, 1.8%), X² (1, n = 530) = 0.66, p = 0.42). No group differences were found for one-month all-cause mortality (Hydro (n = 2, 0.8%) vs. FC (n = 6, 2.1%), X² (1, n = 535) = 1.59, p = 0.29), or incidence of MI (Hydro (n = 0, 0.0%) vs. FC (n = 1, 0.4%), X² (1, n = 535) = 0.89, p = 1.00).

**Table 1:** Guidewire Group Comparisons * Values are mean (standard deviation) ** values are frequency (percentage) HTN – hypertension, DM – diabetes mellitus, CKD – chronic kidney disease, Hx – history, OSA – obstructive sleep apnea, LVEF – left ventricular ejection fraction, CABG – coronary artery bypass graft, CAD – coronary artery disease, CVA – cerebrovascular accident, PPM – permanent pacemaker, MI – myocardial infarction, TAVR – transcatheter aortic valve replacement, p-values for the analyses related to race and BMI are associated with overall group comparisons

Variable/Level	Hydrophilic 0.035 (n = 253)	FC Straight TEF 0.038 (n = 284)	p-value
Age*	77.40 (8.64)	75.12 (9.32)	0.003
Gender (male)**	147 (58.1%)	162 (57.2%)	0.84
Race**			
White	251 (100.0%)	278 (98.2%)	0.09
Black	0 (0.0%)	3 (1.1%)	
Hispanic	0 (0.0%)	1 (0.4%)	
Other	0 (0.0%)	1 (0.4%)	
BMI**			
19–24.9	69 (27.3%)	62 (21.8%)	0.26
25–29.9	82 (32.4%)	84 (29.6%)	
30–34.9	54 (21.3%)	66 (23.2%)	
35–39.9	25 (9.9%)	43 (15.1%)	
40+	23 (9.1%)	29 (10.2%)	
Dyslipidemia**	218 (86.2%)	240 (84.5%)	0.59
HTN**	230 (90.9%)	255 (89.8%)	0.66
DM**	114 (45.1%)	125 (44.2%)	0.84
CKD**	90 (35.6%)	103 (36.3%)	0.87
Smoking**	94 (37.2%)	108 (38.0%)	0.84
OSA**	55 (21.7%)	67 (23.6%)	0.61
LVEF 50% or more**	47 (18.6%)	39 (13.7%)	0.13
Hx of Thrombophilic Disorders**	9 (3.6%)	10 (3.5%)	0.98
Hx of CABG**	33 (13.0%)	35 (12.3%)	0.80
Hx of Native CAD**	117 (46.2%)	122 (43.0%)	0.44
CVA during or past 24 hours**	4 (1.6%)	3 (1.1%)	0.71
CVA within one month of procedure**	4 (1.6%)	2 (0.7%)	0.43
PPM needed within 24–48 hours**	6 (2.4%)	3 (1.1%)	0.32
PPM needed within one month**	7 (2.8%)	5 (1.8%)	0.42
All-cause mortality at one month**	2 (0.8%)	6 (2.1%)	0.29
MI within one month of TAVR**	0 (0.0%)	1 (0.4%)	1.00

## Discussion

Our single-center study found no significant difference in periprocedural CVA rates or other major outcomes between patients with the AV crossed using a 0.035″ hydrophilic wire and those crossed with a 0.038″ fiber-core "straight" guidewire. Among 537 TAVR patients in total, the incidence of stroke was low and similar in both groups at 24 hours and 30 days (p = 0.71 and p = 0.43, respectively). Similarly, one-month rates of new pacemaker implantation, peri-procedural MI, and all-cause mortality did not differ by wire type (all p > 0.29). These findings indicate that guidewire choice did not significantly affect the outcomes in this study.

The overall stroke risk in modern TAVR cohorts is modest but still significant. Contemporary registries report 30‐day stroke rates of about 2-4% [7]. For instance, the Swiss TAVI registry found a 3.0% 30-day stroke rate, with approximately 69% of events occurring within 48 hours of TAVR [7]. Similarly, patient-level pooled analyses show clinical stroke rates of roughly 6-7% by 30 days [8]. However, diffusion‐weighted MRI studies reveal that subclinical “silent” cerebral infarcts happen in most patients [8]. Therefore, while overt stroke is relatively rare, micro‐embolic injury is nearly universal. In this context, the absence of differences in clinically evident CVA in our study aligns with the idea that guidewire type has little impact when compared to other predisposing factors, such as embolic events from extensive AV or LVOT resulting from catheter trauma or during valve deployment, aortic manipulation, and duration of the procedure.

TAVR‐related CVAs are known to be influenced by multiple factors. Procedurally, the primary mechanism is thought to be cerebral embolization of calcific or atheromatous debris during valve crossing and deployment [9]. Following deployment, additional risks include dislodgment of debris from the native valve or aorta, prosthesis‐associated thrombus formation, and new‐onset atrial fibrillation [10]. Factors such as heavy aortic or valve calcification, multiple balloon inflations or device repositioning, and traumatic catheter manipulations have all been related to higher stroke rates [10,11]. In our study, both wire groups likely encountered similar degrees of calcific burden and procedural steps, which may explain the comparable CVA rates. These factors probably overshadow any subtle embolic effect one wire type might have compared to another.

Previous studies specifically addressing guidewire choice in TAVR have focused on technical outcomes rather than stroke. For instance, a recent analysis found that using a very stiff Lunderquist support wire allowed deeper valve implantation depth but did not improve 30-day outcomes, such as conduction disturbances or pacemaker requirement [12]. Similarly, another study of a dedicated TAVR wire reported no wire-related complications such as ventricular perforation or wire displacement in 39 patients [13]. In general, guidewire-related adverse events are rare among experienced operators; as noted in standard interventional manuals, potential hazards include vascular dissection, arrhythmias, entanglement, or ventricular perforation, but these are uncommon when wires are handled carefully. Our data support this notion by showing that, in terms of clinically significant strokes and routine adverse outcomes, wire selection appears benign.

These findings support the practice of tailoring wire choice to operator preference and patient anatomy rather than concern for stroke. Both types of guidewires have theoretical pros and cons (e.g., lubricity versus stability), but we found no evidence that one approach yields better cerebrovascular or clinical outcomes. Operators may thus continue to select wires based on familiarity, perceived ease of valve crossing, or specific anatomical challenges. Importantly, our results do not argue against vigilance; all wires must be manipulated gently to reduce the risk of complications. In the absence of demonstrable benefit, standard operator preference remains reasonable.

Future prospective randomized comparisons of guidewire strategies could validate this study’s findings. Trials incorporating neurological endpoints, such as diffusion‐weighted MRI or transcranial Doppler micro-embolus counts, might uncover differences in subclinical embolic burden between wire types. Exploration of whether certain complex anatomies, such as heavily calcified valves or severe arch atheroma, interact with wire choice in affecting CVA risk would be beneficial. Evaluating cerebral-protection devices in patients stratified by wire type or anatomical risk might also be valuable. Our data suggests that procedural CVA risk management should emphasize careful technique and embolic protection rather than mandating a specific guidewire in TAVR.

Limitations of the study

Retrospective, observational design leaves open the possibility of unmeasured confounding or selection bias. Although baseline characteristics were similar, operators may have subconsciously chosen a given wire for more challenging anatomy, which could influence outcomes. CVA endpoints were based on clinical detection rather than systematic imaging confirmation. We did not perform routine brain imaging, so silent infarcts, which may be related to emboli, were not assessed. This study reflects a single-center experience and may not generalize to all wires, higher-risk patients, or alternative access routes. There was a statistically significant age imbalance between the treatment groups, which may be a source of confounding (multivariable adjustment was not performed). Event rates for several outcomes were very low, leading to limited statistical power and the potential for Type II errors. Non-significant findings should not be interpreted as the absence of an association. These issues reflect the exploratory nature of the analyses, and future studies with larger sample sizes and prespecified confounder adjustments are warranted.

## Conclusions

In summary, our single‐center retrospective analysis of 537 TAVR patients demonstrated that the choice of guidewire, whether hydrophilic coated or not, for crossing the AV does not significantly influence the incidence of clinically overt CVA at 24 hours or one month, nor does it affect secondary endpoints such as new pacemaker implantation, MI, or all‐cause mortality. These findings suggest that, in the setting of modern TAVR techniques where CVA risk is driven predominantly by valve manipulation rather than guidewire‐specific factors, operators may select their preferred wire based on familiarity, anatomical considerations, or ease of crossing without compromising patient safety. Future prospective studies incorporating neuroimaging or micro‐embolus monitoring could further elucidate any subtle subclinical differences, but until such data are available, operator preference and meticulous technique remain the foundations of optimizing cerebrovascular outcomes during TAVR.
